# Effect of Edible Bird's Nest Extract on Lipopolysaccharide-Induced Impairment of Learning and Memory in Wistar Rats

**DOI:** 10.1155/2018/9318789

**Published:** 2018-08-14

**Authors:** S. Careena, D. Sani, S. N. Tan, C. W. Lim, Shariful Hassan, M. Norhafizah, Brian P. Kirby, A. Ideris, J. Stanslas, Hamidon Bin Basri, Christopher Thiam Seong Lim

**Affiliations:** ^1^Department of Medicine, Faculty of Medicine and Health Sciences, UPM Serdang 43400, Selangor Darul Ehsan, Malaysia; ^2^Department of Pathology, Faculty of Medicine and Health Sciences, UPM Serdang 43400, Selangor Darul Ehsan, Malaysia; ^3^School of Pharmacy, Royal College of Surgeons in Ireland, 123 St Stephen's Green, Dublin 2, Ireland; ^4^Department of Veterinary Clinical Studies, Faculty of Veterinary Medicine, UPM Serdang 43400, Selangor Darul Ehsan, Malaysia

## Abstract

Cognitive disability is a common feature associated with a variety of neurological conditions including Alzheimer's Disease (AD), Parkinson's Disease (PD), brain injury, and stroke. Emerging evidence has demonstrated that neuroinflammation plays an important role in the development of cognitive impairment. Current available therapies are relatively ineffective in treating or preventing cognitive disabilities, thus representing an important, unfulfilled medical need. Hence, developing potential treatment is one of the major areas of research interest. Edible bird's nests (EBN) are nests formed by swiftlet's saliva containing sialic acid, which is believed to improve brain function. This present study was embarked upon to evaluate the learning and memory enhancing potential effect of EBN by using Morris water maze test in a Wistar rat model of LPS-induced neuroinflammation. LPS elicited cognitive impairment in the rats by significantly increasing the escape latency while decreasing the number of entries in the probe trial, which are coupled with increased production of proinflammatory cytokines (TNF-*α*, IL-1*β*, and IL-6) and oxidative markers (ROS and TBARS) in the hippocampus. Treatment with EBN (125 mg/kg, 250 mg/kg, and 500 mg/kg; p.o.) effectively reversed the effect of LPS on escape latency and probe trial and, in addition, inhibited the LPS-induced upregulation of proinflammatory cytokines and oxidative markers. These findings are suggestive that there is existence of neuroprotective effect contained inside the edible bird's nest.

## 1. Introduction

EBN or more well known as* “Yan Wo”* among the Chinese population, also, sometimes referred to as* “Caviar of the East”*, is made exclusively from the saliva of cave-dwelling birds and swiftlets from the swiftlet ranches. During the 16th century, EBN was viewed by the Emperors of China as a valuable food with various putative medicinal properties [1]. It is a delicacy produced by the swiftlet species from the genus* Aerodramus*. They build edible nests which are consumed worldwide by humans often as a medicinal food [2]. It has been a symbol of wealth, power, and prestige and being used medicinally in Traditional Chinese Medicine since the Tang and Sung dynasties [3].

EBN contains highly nutritious components like proteins, carbohydrates, mineral salts, and amino acids, as well as being rich in vitamins, hormones, and fatty acids. According to previous findings [2, 4, 5], the highest composition inside the EBN is protein and this is followed by the carbohydrates. A recent study of the unprocessed EBN showed that there was 61% of protein content in EBN samples [6] which was comparable with another study demonstrating that the crude protein content in the red-blood nest was 63% while the white nest was 62% [2]. Protein plays a distinctive role in human body especially in repairing and building body tissues. Carbohydrate components in the EBN are believed to give many therapeutic and medicinal effects to human body. For example, there is 9% of sialic acid in the edible bird's nest which may benefit the neurological and intellectual capacity of infants [7, 8]. Sialic acid possesses anti-inflammatory property which can modulate a wide variety of physiological and pathological processes. Sialic acid is generally found glycosidically linked to form N-acetylgalactosamine (GalNAc), a compound which is important in proper functioning of synapses. GalNAc also has been shown to be able to improve memory [9]. Until today, there has been no scientific evidence to prove the link between sialic acid in EBN and cognitive function. Hence, this present study was embarked upon to evaluate the learning and memory potential effect of EBN using Morris water maze in a Wister rat model of LPS-induced neuroinflammation. LPS elicit cognitive impairment in the rats by significantly increasing the escape latency while decreasing the number of entries in the probe trial [10].

## 2. Materials and Methods

### 2.1. Chemical/Reagents

Pepsin from porcine stomach mucosa and pancreatin enzyme was purchased from Nacalai Tesque Inc. Kyoto, Japan. Kit for protein assay was purchased from Thermo scientific Ltd. Sodium hydrogen carbonate, sodium bicarbonate, hydrochloric acid, and sodium hydroxide were purchased from Sigma-Aldrich (St. Louis, USA).

### 2.2. Sample Collection

Edible bird's nest was collected from seven different regions (East Malaysia, northern region, west coast, southern region, heavily polluted industrial area, and east coast of Peninsular Malaysia) around Malaysia and all of them were given a code and blinded to the researchers who carried out the experiments (Table 1)

### 2.3. Processing and Preparation of Aqueous Extract of EBN

EBN was extracted as per the method with slight modification [11]. Briefly, about 1 g of the EBN was crushed to powder using pestle and mortar. The powdered sample was then collected, mixed with 100 mL of ultrapure water in a conical flask. The mixture was mixed thoroughly, double boiled at 100°C in water bath for 1 h (according to the Chinese technique of cooking to preserve the taste) and then filtered through a muslin cloth. The extract (filtrate) was subsequently frozen in −80°C and freeze-dried into dry powder in a freeze dryer and later stored at −20°C until required for further use.

### 2.4. Sample (Extract) Digestion

The* in vitro* digestion of the freeze-dried EBN extract was carried out using pepsin-pancreatin. Briefly, the aqueous extract was adjusted to pH 2.0 with 1.0 M HCL. Pepsin was then added and the mixture was incubated at 37°C for 2 hours in a shaking water bath. Subsequently, the pH value was adjusted to 5.3 with 0.9 NaHCO_3_ solution and later to 7.5 with 1.0 M NaOH. Thereafter, pancreatin was added and the mixture was reincubated at 37°C for 2 hours. The reaction was terminated by keeping the test tube boiling for another 10 minutes. The mixture was cooled at room temperature, filtered, freeze-dried, and finally stored at −20°C until use.

### 2.5. Sialic Acid Analysis: HPLC with Fluorescence Detection

For the sialic acid analysis of the EBN samples, we sent our samples to Sani Chem Resource Centre, a certified laboratory. Briefly, 5 mg of EBN freeze-dried sample was dissolved in 1 ml of 0.5 mol/L of sodium hydrogen sulphate solution and kept in 80°c water bath. After cooling, the derivatization was carried out using O-phenylenediamine. The chromatographic separation was achieved on a C18 column using a mobile phase that composed of 1.0% tetrahydrofuran aqueous and acetonitrile at a flow rate of 1.0 ml/min in the isocratic elution mode. The fluorescence detector was used with excitation wavelength of 425 nm and emission wavelength of 425 nm. Quantitation results were obtained from external curve of N-acetylneuraminic acid [12].

### 2.6. Animal Handling and Housing

Male Wistar strain rats, 12-14 weeks of age and weighing between 250 and 300g, were utilized for this study. The animals were procured from Takrif Bistari Enterprise, No. 5, Jalan 1/4 Taman Kembangsari, 43300, Seri Kembangan, Selangor, Malaysia. All rats were housed in groups in stainless steel cages for a period of 10 days to acclimatise to laboratory environment at an ambient temperature of 25±2°C with 12 h light: 12 h dark cycle. They were fed on standard commercial rat/mouse pellet diet (Specialty feeds, Glen forest, Western Australia) and water was provided* ad libitum* prior to the investigation. All animal experiments were conducted in accordance with the principles of laboratory animal care specified and approved by Universiti Putra Malaysia Animal Care Use Committee (UPM/IACUC/AUP-R064/2014)

### 2.7. Experimental Animal

Forty-eight animals were used for the study and randomly divided into six groups of eight rats each: Group I, normal control; Group II, LPS-induced rats without treatment as negative control; Group III-V, LPS- induced rats pretreated with graded doses (125, 250, and 500 mg/kg, respectively) of EBN; Group VI, LPS-induced rats pretreated with standardized ginkgo biloba extract, EGb 761 (Tanakan R, 40 mg/kg, IPSEN industry, 28100 Dreux, France), as positive control.

### 2.8. EBN, EGb, and LPS Treatments

EBN extract as prepared above was diluted to achieve graded doses (125, 250, and 500 mg/kg, respectively) and fed orally to rats (Groups III to V) for seven days prior to intraperitoneal (i.p.) injection of 1 mg/kg LPS (Escherichia coli serotype: 055:B5; Sigma-Aldrich, St. Louis, MO) which was dissolved in saline. Similarly, EGb 761 (200 mg/kg) was prepared and fed orally to the rats in Group 6 prior to LPS injection.

### 2.9. Morris Water Maze

The Morris water maze is a behavioral test for studying spatial learning and memory in animals. Rats were tested for cognition function using a 2-day water maze protocol with slight modification [13]. Briefly, following pretreatment with EBN/EGb for 7 days, the experimental rats were injected with LPS on the 8th day. The MWM training day took place on Day 8 (prior to administration of LPS), with testing conducted 24 h later (hidden platform trials). Data including swim latency, path length, and swimming speed during the trials will be recorded using ANY-maze Video Tracking System (Stoelting, Wood Dale, IL, USA), connected to a CCD camera. All data were thereafter used to assess performance in the MWM task. Similarly, latency to enter and the time spent in the novel quadrant during the probe trial were also evaluated.

### 2.10. Sample Collection

At the end of the behavioral experiment (Day 3), all the rats were sacrificed via the use of CO_2_ and the brain harvested. The hippocampus was immediately frozen in liquid nitrogen and then transferred to −80°C until required for analysis.

### 2.11. Measurement of Proinflammatory Cytokines

Proinflammatory cytokines (TNF-*α*, IL-1*β*, and IL-6) from the brain sample supernatant were estimated using commercial ELISA kits (Cusabio, Wuhan, China) according to the manufacturer's instructions.

### 2.12. Determination of Oxidative Stress Mediators

#### 2.12.1. Determination of Reactive Oxygen Species (ROS) Levels

Production of intracellular ROS in the brain section lysates was determined using 2,7-dichlorofluorescin diacetate (DCF-DA) according to the method earlier described by Shinomol and Muralidhara [14] with a slight modification. Briefly, reaction mixture (50 *μ*l sample and 100 *μ*l (DCF-DA 0.5M)) was incubated at room temperature for 30 min and the DCF product was measured using fluorescence spectrophotometer at 484 nm excitation and 530 nm emission. ROS generation was reported as fold change against control.

#### 2.12.2. Determination of Lipid Peroxidation

Estimation of lipid peroxidation level was determined according to the MDA index using thiobarbituric acid reactive substance (TBARS) assay based on the original method described by Draper & Hadley [15] with a slight modification. Briefly, 200 *μ*l of 20% trichloroacetic acid (TCA) was mixed with 50 *μ*l brain section homogenate and then centrifuged at 4 × 10^3^ g over 20 min. Equal volumes of thiobarbituric acid (TBA) reagent and supernatant were then mixed together. The content of each sample was then heated using a water bath at 95°C for 10 min and cooled and optical density was measured spectrophotometrically at 532 nm (VersaMax, Molecular devices, US).

### 2.13. Statistical Analysis

Results were expressed as mean ± standard deviation (SD) using Microsoft Excel 2010. Data were further analysed with one-way analysis of variance (ANOVA). Statistical differences were considered significant at p<0.05.

## 3. Results

### 3.1. Sialic Acid Analysis

The sialic acid analysis of the EBN was performed by Sani Chem Resource using their in-house HPLC method. In our studies, there is presence of different amount of sialic acid found in EBN from different regions in Malaysia. The content of the sialic acid increases after enzyme digestion. EBN 02 has the highest content of sialic acid for the EBN crude preparation while EBN 04 showed the highest content of sialic acid for EBN enzyme digested preparation (Table 2).

### 3.2. Pretreatment Experimental Studies

**Figure 1:**
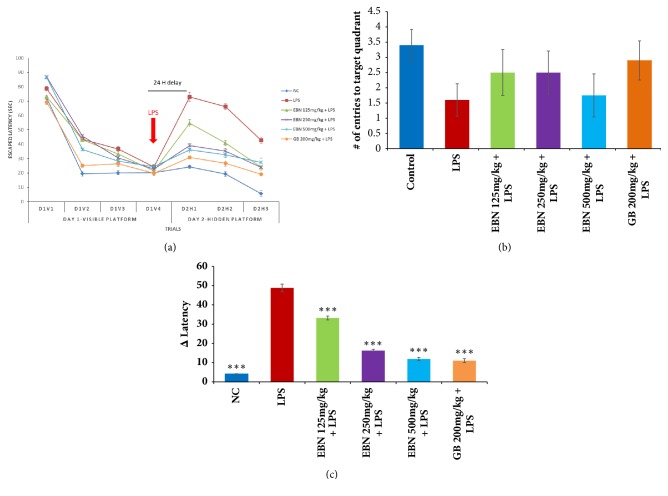
(a) Morris water maze escape latency test. EBN: edible bird's nest; GB: ginkgo biloba; LPS: lipopolysaccharide.). The results are presented as mean ± SD of each group (n=8). GB: ginkgo biloba; LPS: lipopolysaccharide; D: day; V: visible; H: hidden). (b) Morris water maze test escape latency difference. (c) Morris water maze probe trial test.

For pretreatment, rats that were treated with LPS showed significant spatial memory deficits in a 2-day water maze protocol. The visible platform performance trials (Figure 1(a)), latency to reach the platform, and the difference in latencies significantly increased in the LPS group compared to normal control group. Correspondingly, the number of entries (Figure 1(b)) into the quadrant that previously contained the escape platform was significantly less in the LPS-treated group compared with vehicle-treated control animals as revealed by the probe trial. EBN treatment dose-dependently reduced latency (Figure 1(c)). A similar effect was displayed by ginkgo biloba (GB), which was used as a positive control.

Performance assessment of the effect of EBN and GB extracts against LPS-induced cognitive impairment in rats using the Morris water maze (MWM) task was carried out. Figure 1(a) shows the escape latency of the animal travelled from the starting point to reach the submerged platform. The results showed that the escape latencies between Day 1, visible platform trial 1, and Day 2, hidden platform trial were significantly (*∗∗∗*P≤0.001) decreased in the treated groups compared with corresponding trial of LPS group. The latency differences (D2H1-D1V4) were also significantly (*∗∗∗*P≤0.001) lower in the treated groups compared with LPS group (Figure 1(b)). As for the probe trial (number of entries into previously contained quadrant), it showed no significant difference between the treated and LPS groups (Figure 1(c)).

### 3.3. Effect of Pretreatment with EBN on LPS-Induced Hippocampal Production of Proinflammatory Cytokines

**Figure 2:**
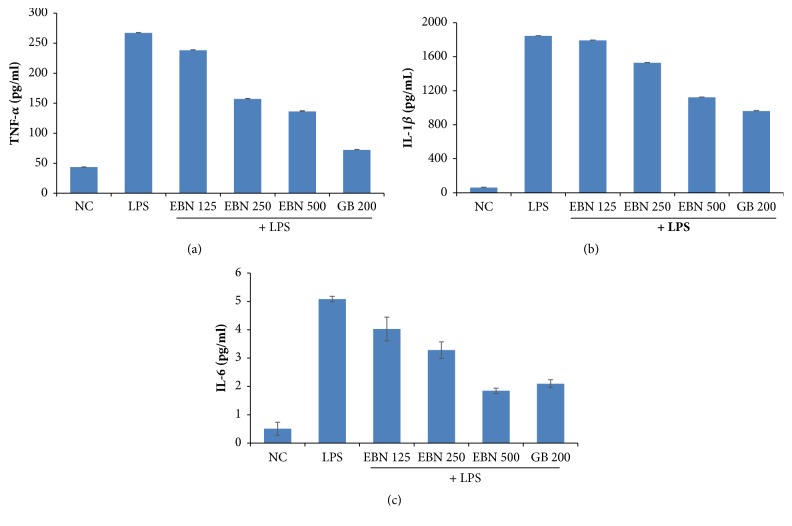
(a) Effect of pretreatment of EBN and TNF levels. (b) Effect of pretreatment of EBN and IL-1B levels. (c) Effect of pretreatment of EBN and IL-6 levels.

As shown in Figures 2(a), 2(b), and 2(c), LPS (1 mg/mL, i.p.) administration caused increased production of TNF-*α*, IL-1*β*, and IL-6. However, EBN pretreatment significantly inhibited the production of these inflammatory cytokines (*∗*P < 0.05).

### 3.4. Effect of EBN Pretreatment against LPS-Induced Hippocampal Production of Oxidative Stress Mediators

**Figure 3:**
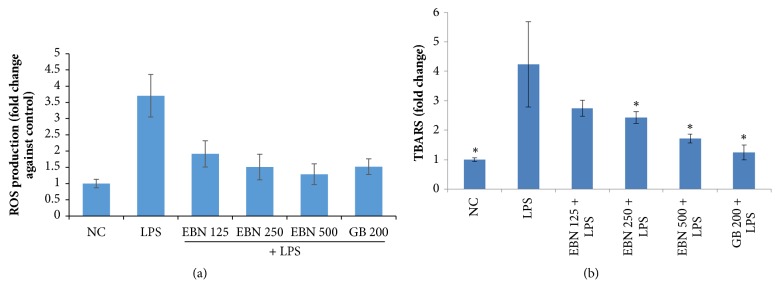
EBN significantly inhibited LPS-induced generation of oxidative stress mediators, (a) ROS and (b) TBARS.

Administration of LPS caused marked increase in the productions of ROS and TBARS (Figure 3). However, pretreatment with EBN prior to LPS administration significantly attenuated the production of these molecules in a dose-dependent manner.

## 4. Discussion

Sialic acid, one of the key components of EBN, has been proposed to have an effect on cognition and neurodevelopment. Our study has demonstrated that EBN can dose-dependently protect against LPS-induced neuroinflammation and reverse LPS-induced memory impairment.

We also found that the content of sialic acid varied among the regions in Malaysia. The reason may be due to seasonal variations. It has been demonstrated that the highest quality of EBN was produced during the early and late rainy seasons [6]. The unprocessed EBN samples harvested during the rainy seasons gave higher composition of proteins as compared to those collected during the nonrainy seasons [6]. It is proposed that the differences in nutritional compositions might be affected by the locations and breeding sites of the swiftlets [16].

Alzheimer's disease (AD) is a neurodegenerative disease of the central nervous system (CNS) and it is the most common form of dementia [17]. Also, it resulted in the development of various cognitive deficiencies especially the memory impairment.

Studies have long shown that inflammatory processes in the brain is associated with impaired cognitive function [18]. AD patients demonstrate a gradual development of forgetfulness and some abnormalities in language and speaking ability [18]. Study showed that there are approximately 4.6 million people of new cases who suffered from AD or dementia related disease. It is predicted that by the year 2030, the numbers of cases will be doubled [19]. Currently there is approximately 44 million of people worldwide to have AD or related dementia [19]. AD also reported to affect one out of nine adults at age 65 and above based on the figures provided by Alzheimer's Association in 2013. In Malaysia, it is estimated that there are approximately 50000 people affected by AD. The risk of developing AD increases with age.

In this study, rats treated with LPS showed deficit in memory and the hippocampus of these animals had increased levels of IL-1*β*, TNF-*α*, and IL-6 compared to the control group. This is consistent with recent studies that systemic administration of LPS alone caused deficit in learning and memory and this is associated with increased levels of brain proinflammatory mediators including TNF-*α* and IL-1*β*. [20–24]. However, pretreatment with EBN significantly prevented this impairment induced by LPS and inhibited the production of these cytokines dose-dependently. This outcome is in parallel with the study of Kahn [21]. In addition, LPS has also been reported to elicit oxidative stress processes besides neuroinflammation [22]. The elevated cytokine levels in this study could explain the cognitive deficit observed in the LPS-treated group. These findings agree with recent studies that reported LPS caused upregulation of IL-1*β*, IL-6, and TNF-*α* in the hippocampus resulting in neuroinflammatory pathologies [24].

The ability of EBN to protect the hippocampus from inflammatory damage could be explained in part due to presence of sialic acid. This is supported by the fact that sialic acid has been shown to enhance the intellectual capacity and also neurological functional of the infant [8]. There is a total of 9% of sialic acid of the total carbohydrates composition in EBN [7, 8]. Sialic acid is known to improve memory as it acts as an agent for the improvement of synaptic function, mediation of ganglioside distribution, and structure in the brain [25, 26]. The anti-inflammatory effect by EBN in this study may be attributed to sialic acid content. There were several studies reported that sialic acid modulates the anti-inflammatory activities [27, 28]. It has been reported that the application of sialic acid is important and beneficial to medical field especially in mediate or modulate a wide variety of physiological and pathological processes [29]. However, for the posttreatment results, EBN failed to show neuroprotective effect towards induced rats. This indicates that prevention is better than cure; a good strategy to prevent development of AD could be achieved through consumption of food with medicinal values. As EBN have been widely used and consumed for various health benefits reasons, we have provided preliminary evidence that this valuable delicacy may have a role in preventing the development of AD.

## 5. Conclusion

This study revealed that geographical factor plays an important role on the nutritional quality of the EBN and that neuroinflammation is crucial in the pathology associated with cognitive impairment. Overall, anti-inflammatory property by EBN was shown significantly in EBN01. This clearly indicates that demography of EBN source plays a role in producing high quality EBN with neuroprotective property. Moreover, sialic acid from EBN could be useful as health enhancing functional food against neurodegenerative diseases. Finally, it is suggested that EBN significantly enhanced memory and confirmed potent neuroprotection through inhibition of neuroinflammatory and oxidative stress processes.

## Figures and Tables

**Table 1:** Coded EBN according to the regions where the nests were harvested.

**EBN CODE**	**ORIGIN**
EBN 01	East Coast of Peninsular Malaysia
EBN 02	East Malaysia
EBN 03	Southern region of Peninsular Malaysia
EBN 04	Heavily polluted industrial area
EBN 05	Northern region of Peninsular Malaysia
EBN 06	West coast of Peninsular Malaysia
EBN 07	East Coast of Peninsular Malaysia

**Table 2:** Sialic acid analysis of EBN from different regions.

**SAMPLE**	**CRUDE EBN** **CONTENT (**%**)**	**DIGESTED EBN** **CONTENT (**%**)**
EBN 01	5.76	9.24
EBN 02	11.00	13.2
EBN 03	5.69	5.34
EBN 04	7.87	14.9
EBN 05	9.67	7.07
EBN 06	9.23	9.71
EBN 07	5.47	5.24

## Data Availability

The data used to support the findings of this study are available from the corresponding author upon request.

## Abbreviations

EBN: Edible bird's nest
AD: Alzheimer's Disease
PD: Parkinson's Disease
LPS: Lipopolysaccharide
GalNAc: N-acetylgalactosamine
TBARS: Thiobarbituric acid reactive substance
GB: Ginkgo biloba
ROS: Reactive oxygen species
IL: Interleukin
TNF: Tumour necrosis factor.
